# Crystal Structure of *Deinococcus radiodurans* RecQ Helicase Catalytic Core Domain: The Interdomain Flexibility

**DOI:** 10.1155/2014/342725

**Published:** 2014-08-27

**Authors:** Sheng-Chia Chen, Chi-Hung Huang, Chia Shin Yang, Tzong-Der Way, Ming-Chung Chang, Yeh Chen

**Affiliations:** ^1^Department of Biotechnology, Hungkuang University, Taichung 433, Taiwan; ^2^Taiwan Advance Biopharm (TABP), Inc., Xizhi City, New Taipei City 221, Taiwan; ^3^Institute of Biochemistry, National Chung-Hsing University, Taichung 40227, Taiwan; ^4^Department of Biological Science and Technology, College of Life Sciences, China Medical University, Taichung 40402, Taiwan; ^5^Department of Nutrition, Hungkuang University, Taichung 433, Taiwan

## Abstract

RecQ DNA helicases are key enzymes in the maintenance of genome integrity, and they have functions in DNA replication, recombination, and repair. In contrast to most RecQs, RecQ from *Deinococcus radiodurans* (DrRecQ) possesses an unusual domain architecture that is crucial for its remarkable ability to repair DNA. Here, we determined the crystal structures of the DrRecQ helicase catalytic core and its ADP-bound form, revealing interdomain flexibility in its first RecA-like and winged-helix (WH) domains. Additionally, the WH domain of DrRecQ is positioned in a different orientation from that of the *E. coli* RecQ (EcRecQ). These results suggest that the orientation of the protein during DNA-binding is significantly different when comparing DrRecQ and EcRecQ.

## 1. Introduction

RecQ helicases are a family of DNA strand-separating enzymes conserved from bacteria to humans that play a crucial role in the maintenance of genome stability [1]. Bacteria have only one RecQ homolog, whereas higher eukaryotic organisms express multiple homologs of RecQ enzymes. Five members of the RecQ family have been found in humans, namely, RECQ1, BLM, WRN, RECQ4, and RECQ5, and defects in three of the five RecQ family members are related to human disorders. Specifically, mutations in the human genes BLM, WRN, and RECQ4 cause Bloom's syndrome, Werner's syndrome, and Rothmund-Thomson syndrome, respectively. These mutations result in genomic instabilities and a predisposition to cancer [2, 3]

Most RecQ proteins share a helicase catalytic core and a C-terminal helicase-and-RNase-D-C-terminal (HRDC) domain. The minimal helicase catalytic core consists of two RecA-like domains, a Zn-binding domain (ZBD), and a winged-helix (WH) domain. Each individual domain has its own function and structure [4]. The helicase catalytic core is crucial for the ATPase and DNA-unwinding activities of these RecQ proteins. The HRDC domain is responsible for regulating DNA-binding affinity with a wide variety of DNA substrates [5]. To date, tertiary structures of the RecQ helicase catalytic core have been reported and determined by X-ray crystallography, including those of* E. coli* RecQ (PDB 1OYW) and human RecQ1 (hRecQ1) (PDB 2V1X) [4, 6]. These structures have been observed to adopt similar domain architectures, forming Y-shaped molecules. However, the hairpin structures and domain orientation of the WH domains, which are important for DNA binding and unwinding, are markedly distinct between the EcRecQ and the hRecQ1.

*Deinococcus radiodurans* is best known for extraordinary resistance to ionizing radiation, ultraviolet (UV) radiation, and chemical mutagens. This bacterium can survive under conditions of 7000 kGy of ionizing radiation with only 10% cell death; a radiation dose of greater than 50 Gy is lethal to most organisms [7]. Over 100 double-strand breaks (DSBs) can be mended throughout its genome during a postirradiative incubation [2, 8]. Because of its remarkable ability to repair DNA damage,* D. radiodurans* can become an excellent model system for studying the mechanisms of DNA repair. In this bacterium, RecD is the only member that is identified in the RecBCD pathway, whereas all of the members associated with the RecFOR pathway have been found in the genome of* D. radiodurans*, suggesting that the RecFOR pathway is the main repair pathway of DNA damage in this organism [9].

*D. radiodurans* has a unique RecQ homolog with three HRDC domains (HRDC1, HRDC2, and HRDC3) at its C terminus. The function of DrRecQ has been characterized, confirming that the unusual domain arrangement of DrRecQ has the extraordinary ability to repair DNA damage in* D. radiodurans *[10]. HRDC1 and HRDC3 have also been structurally characterized [5, 11]. However, the structure of the helicase catalytic core remains undetermined. To better understand the structural and functional relationships of DrRecQ, we present crystal structures of the minimal helicase catalytic core from DrRecQ and its binary complex with ADP. Surprisingly, superposition of the apoenzyme against the binary complex reveals that the WH domain is positioned at a different orientation in these two structures, indicating the flexibility of the WH domain interdomain. Owing to the importance of the WH domain for DNA binding, its flexibility is likely involved in regulating DNA recognition. A comparison of DrRecQ and EcRecQ reveals that the domain orientation of the WH domain is significantly different between these two proteins, suggesting that the orientation of the DNA-binding motif is distinct between the DrRecQ and the EcRecQ.

## 2. Materials and Methods

### 2.1. Cloning, Expression, Purification, Crystallization, and Data Collection of DrRecQ

The gene cloning, protein expression, and purification of DrRecQ have been previously reported [12]. DrRecQ was crystallized using the sitting-drop vapor diffusion method with a buffer containing 0.1 M HEPES (pH 7.7), 14% PEG 8 K, and 8% ethylene glycol. It formed orthorhombic crystals with the following cell dimensions: *a* = 85.7 Å, *b* = 98.5 Å, and *c* = 152.8 Å, with two molecules per asymmetric unit. All the DrRecQ crystals were cryoprotected with 25% glycerol added to the reservoir solution before flash cooling in a stream of nitrogen at 100 K. Crystals of DrRecQ diffracted at 2.8 Å at the National Synchrotron Radiation Research Center (NSRRC) BL13C1 in Taiwan. DrRecQ in complex with ADP was crystallized with a buffer containing 0.1 M imidazole (pH 7.2) and 16% PEG 20 K. The final crystals were orthorhombic with the following cell dimensions: *a* = 84.7 Å, *b* = 95.6 Å, and *c* = 183.8 Å, with two molecules per asymmetric unit. The crystal of the binary complex diffracted to a resolution of 2.9 Å at NSRRC BL13C1.

### 2.2. Structure Determination

The crystal structure of the helicase catalytic core of DrRecQ was solved using the molecular-replacement method and the Phaser program with the coordinates of EcRecQ derived from a search model [13]. The crystal structure of the DrRecQ-ADP complex was solved using the molecular replacement program Phaser and the structure of the apoform DrRecQ was derived from a search model. The structure was completed via multiple manual interactions in COOT [14]. Structure refinement was performed by using the Phenix program and refined with tight NCS restraints [15]. The quality of all models was assessed using the Molprobity program [16]. Stereochemical libraries were prepared using the Phenix program elBOW. The statistical analysis of the data collection and refinement is summarized in Table 1. The coordinates and structural factors of the DrRecQ and DrRecQ-ADP crystals have been deposited in Protein Data Bank with accession codes 4Q48 and 4Q47, respectively. The structural figures were prepared in the PyMOL program (The PyMOL Molecular Graphics System, Version 0.99rc6, Schrödinger, LLC.).

## 3. Results and Discussion

### 3.1. Overall Structure

**Figure 1:**
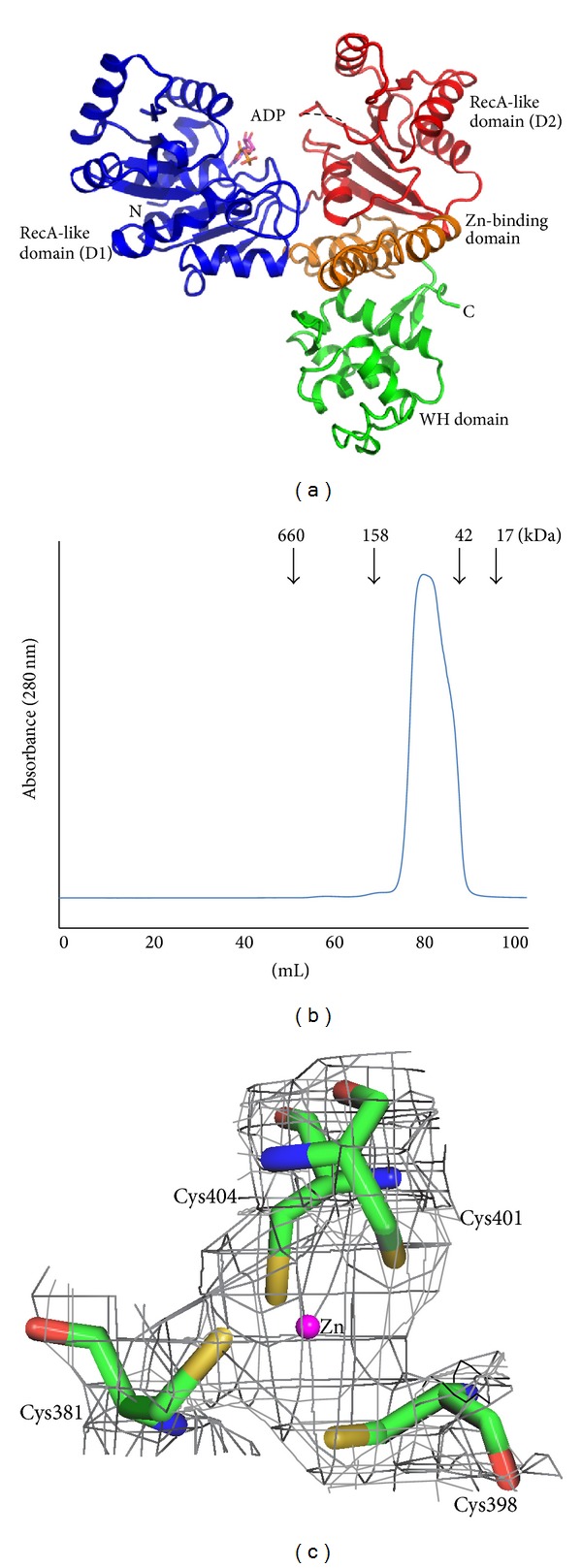
(a) Crystal structure of the* D. radiodurans* RecQ catalytic core. The two RecA-like domains, D1 and D2, are colored in blue and red, respectively. The zinc-binding domain is highlighted in orange and the WH domain in green. ADP (magenta) is depicted in stick representation. (b) The size exclusion chromatography profile of DrRecQ. (c) The zinc-binding site of DrRecQ. The four conserved cysteine residues are shown in stick and a zinc ion is colored in magenta. The 2F_o_-F_c_ electron density map contoured at the 2*σ* level is colored in gray.

The crystal structure of the helicase catalytic core of RecQ from* D. radiodurans* was solved using the molecular-replacement method with the coordinates of EcRecQ from a search model and was refined at a resolution of 2.80 Å. The apoenzyme structure was subsequently used to solve the binary complex structure with ADP at resolution of 2.90 Å. In the binary complex structure, residues 5–517 of the polypeptide chain were well defined in the electron-density map; the exception was the 4 N-terminal end residues and residues 296–300, which were not visible. A ribbon representation of a monomer is shown in Figure 1(a). Both crystal forms contain two molecules in each asymmetric unit. Gel filtration analysis showed that the helicase catalytic core exists as a monomer in solution (Figure 1(b)). Thus, we consider the monomeric structure the biological unit.

The crystal structure reveals that the helicase catalytic core of DrRecQ is composed of four domains. The helicase catalytic core of DrRecQ contains four domains assembled in a trilobed or Y-shaped manner, with major clefts on its surface. The N-terminal part contains the common core structure of the RecQ helicase family, which consists of two canonical RecA-like domains, D1 (amino acids 1–207) and D2 (amino acids 208–341), that are each composed of a central *β*-sheet surrounded by α-helices. Highly conserved signature sequence motifs are located in a cleft at the interface of D1 and D2. These motifs are typically involved in the hydrolysis of ATP and nucleic acid binding. The following Zn-binding domain (amino acids 342–407) possesses a Zn-binding motif and two antiparallel helices. The electron density maps of the Zn-binding motif reveal that the zinc ion is tetrahedrally coordinated by the side chains of four conserved cysteine residues (Cys381, Cys398, Cys401, and Cys 404) (Figure 1(c)). The zinc ion seems to be dispensable for the stabilization of the protein structure [4]. The C-terminal region adopts a WH fold (amino acids 407–517) and is structurally homologous to similar folds in WRN and RecQ1, even though they share very little sequence conservation. The WRN WH-DNA complex structure (PDB code 3AAF) showed that the WH domain directly participates in DNA binding and base pair separation [17]. Thus, the WH fold of DrRecQ was considered to be a DNA-binding domain for the recognition of different DNA substrates.

### 3.2. DrRecQ-ADP Interactions

**Figure 2:**
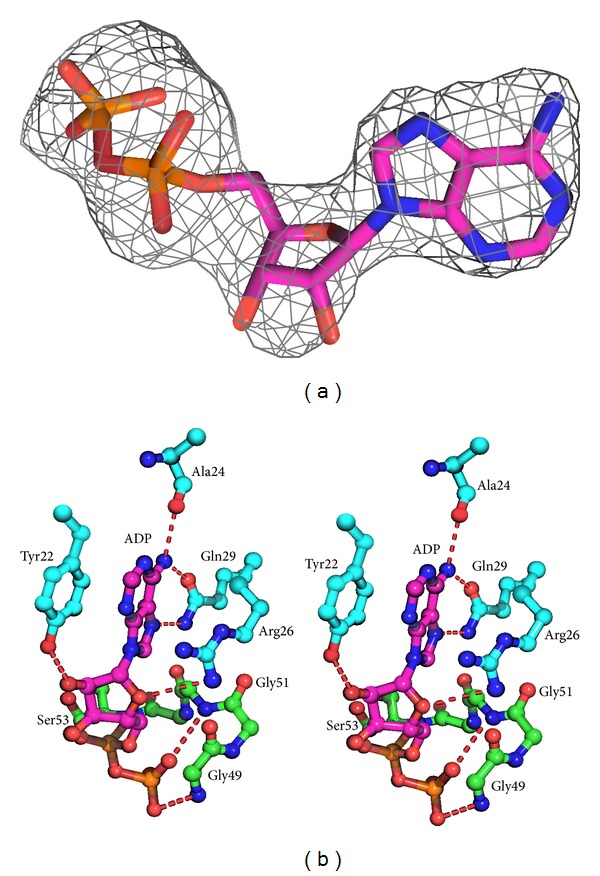
(a) The electron density for ADP from F_o_-F_c_ map seen at 3*σ*. (b) Stereo view of the catalytic site in the DrRecQ catalytic core. The residues that interact with ADP in DrRecQ are shown as ball-and-stick models (magenta). Main chain and carbon are colored according to the conserved motifs: motif 0 (cyan) and motif I (green).

**Figure 3:**
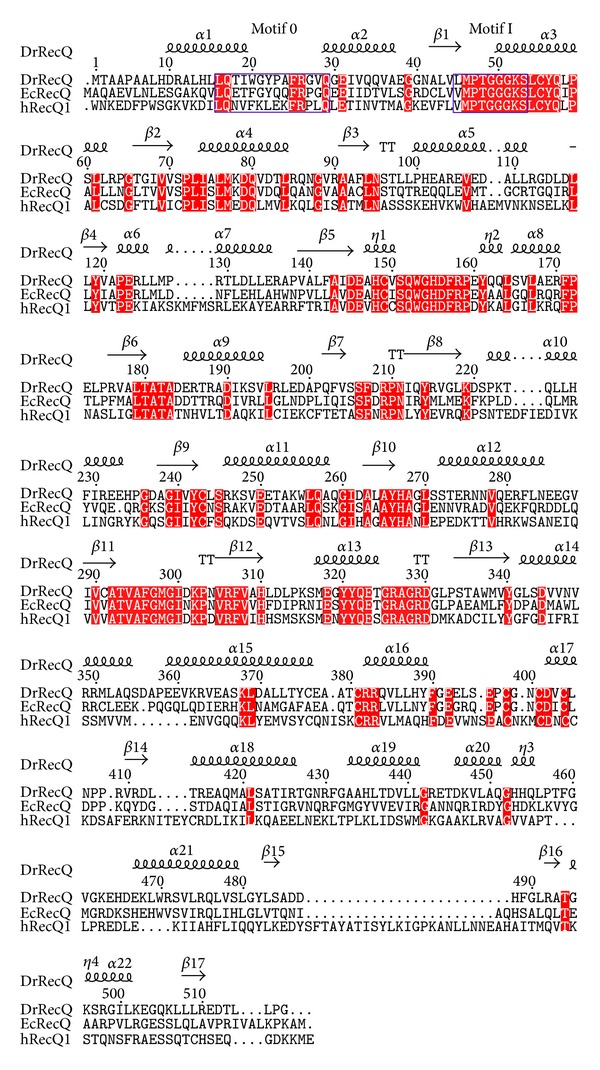
Sequence alignment of the catalytic core from various RecQ proteins. Conserved helicase motifs 0 and I are labeled and enclosed in blue boxes. The conserved residues are shaded in red.

The nucleotide-binding pocket surrounded by conserved motifs is located at the D1 domain. Inspection of the electron density map revealed that electron density is strong in the pocket. Although we initially tried to model ATP into the experiment map, the *γ*-phosphate cannot fit well, suggesting that the ATP has been hydrolyzed to ADP during crystallization despite not adding a magnesium ion (Figure 2(a)). ADP is bound with extensive interactions with the protein (Figure 2(b)). The adenine moiety is sandwiched between Arg26 and Tyr22. The N6 and N7 atoms of the adenine ring are hydrogen-bonded to the side chain of Gln29, and the N6 atom is also situated within hydrogen-bonding distance of the main chain carbonyl oxygen atoms of Ala24. The ribosyl moiety is anchored via hydrogen bonds with the C-2 hydroxyl group of the side chain of Tyr22. The diphosphate is bound by backbone amides of motif I. ADP binding to the D1 domain causes slight alterations in motif I. The sequence alignment shows that motifs 0 (amino acids 16–29) and I (amino acids 45–53) are highly conserved in three RecQ proteins (Figure 3). All of the nucleotide-interacting residues of DrRecQ are conserved in EcRecQ and the ATPase-active conformation of DrRecQ is similar to those of other RecQ family members.

### 3.3. Structural and Sequence Comparisons

**Figure 4:**
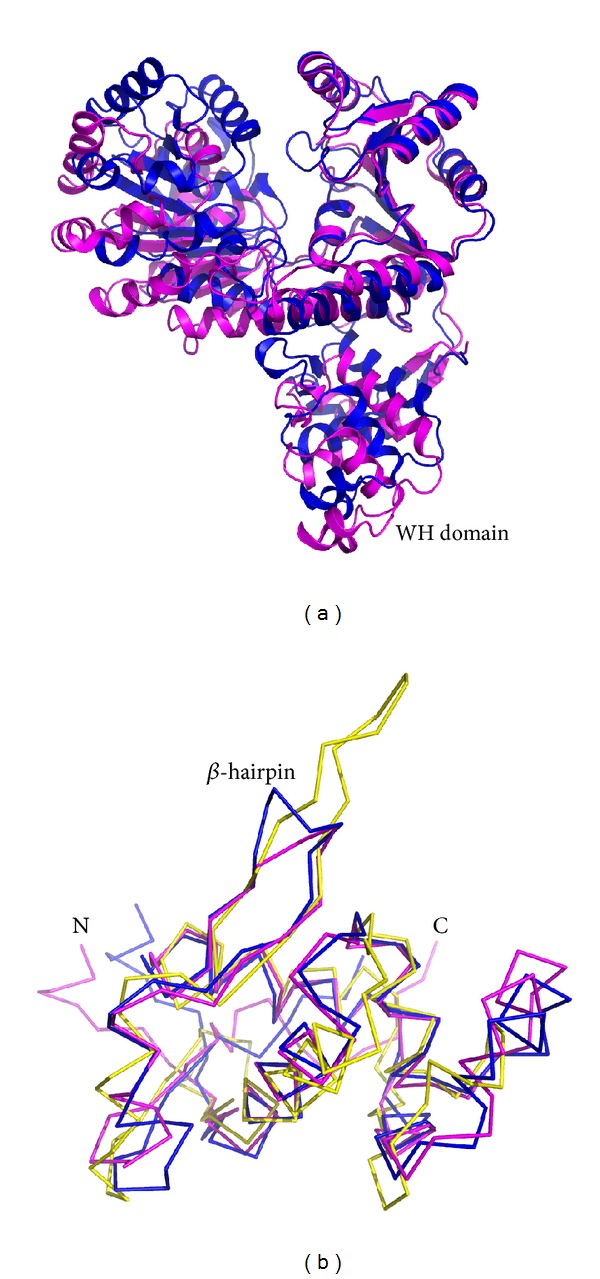
(a) Overlay of DrRecQ (magenta) and EcRecQ (blue). Structures were superposed using the RecA-like 2 domain as a reference. (b) Structure comparison of *β*-hairpins between RecQ members. The DrRecQ (magenta) is shown overlaid with human RecQ1 (yellow) and with* E. coli* RecQ (blue).

Structural similarity searches with Dali revealed that DrRecQ is similar to other RecQ proteins [18]. The best hits include EcRecQ and hRecQ1. Most of the secondary structural elements in DrRecQ and EcRecQ are highly conserved. The percentages of sequence identity between DrRecQ with EcRecQ and hRecQ1 are 49% and 33%, respectively. The rmsd values of superimposition between DrRecQ with EcRecQ and hRecQ1 are 3.7 and 10.8 Å for 498 and 474 C_*α*_, respectively. These data indicate that DrRecQ is more structurally similar to EcRecQ and more distant from hRecQ1. The domains of DrRecQ could individually superimpose well with both EcRecQ and hRecQ1, but the high rmsd value may be due to difference in the interdomain orientations between the structures. Comparison of the three structures demonstrates that there is conservation of domain architecture, specifically in the N-terminal portions of two RecA-like domains, the following Zn-binding domain and the C-terminal WH domain. A structural alignment was carried out for representing structures using the Pymol program with the resulting structural superimposition shown in Figure 4(a). These structures were superimposed using the D2 domain as reference, showing that the relative orientation of the WH domains is dramatically different. These results suggest that orientation of the DNA-binding motif is distinct when comparing DrRecQ and EcRecQ. Additionally, several studies have shown that the *β*-hairpin structures in the WH domains of the human RecQ members are necessary for DNA unwinding, while mutational analysis shows that H491 in the *β*-hairpin of EcRecQ is not required for helicase activity, suggesting a different mode of action between the bacterial and human RecQs [6]. A structural comparison of DrRecQ, hRecQ1, and EcRecQ reveals that the prominent *β*-hairpin of DrRecQ is shorter than hRecQ1 but resembles that of EcRecQ (Figure 4(b)). This may indicate that the functional role of *β*-hairpin of DrRecQ is similar to that of EcRecQ.

### 3.4. Interdomain Flexibility of DrRecQ

**Figure 5:**
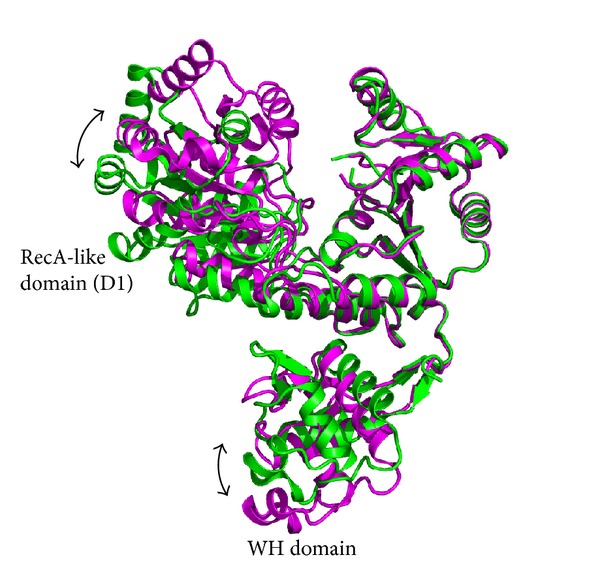
Interdomain flexibility of DrRecQ. The apoform is colored in magenta and the ADP-bound form in green. The structures are superimposed by using the RecA-like 2 domain as a reference.

A structural overlay of the free and ADP-bound forms of DrRecQ reveals that the two structures differ in the relative positions and orientation of D1 and WH domains (Figure 5). A structural shift in the D1 and D2 domains indicates that a high degree of interdomain flexibility occurs. The motion may represent an interdomain motion required for interaction with DNA, though the detailed mechanism of DNA unwinding is still unclear. Moreover, the structural differences between the WH domains of the two forms are well demonstrated by the rigid-body movement between the Zn-binding and the WH domains. This relative domain orientation of DrRecQ was further analyzed using Chimera software [19]. The WH domain rotates 24° toward the Zn-binding domain. Because the WH domain is not close to the nucleotide-binding pocket, differences in the orientation of the WH domain do not appear to be induced by ADP binding. This result is most likely due to DrRecQ crystallized in different conditions. The distinct domain orientation also indicates the interdomain flexibility of the WH domain. This motion generates a closed conformation in the binary complex structure and slightly enlarges the cleft between Zn-binding and WH domains in the apoform structure. To the best of our knowledge, the WH domain plays an important role in DNA binding and unwinding, even though the bacterial RecQ-DNA complex structures that define how protein-DNA binding occurs are unavailable. The motion of the WH domain in DrRecQ may allow the WH domain to easily interact with different DNA substrates or to cope with HRDC domains as the DNA is unwinding, which seems to contribute to the remarkable ability of repairing DNA damage in this extremophilic organism. Moreover, the orientation of the DrRecQ WH domain is different than that observed in both EcRecQ and hRecQ1, which may reveal a functional difference in DNA binding and unwinding. The interdomain flexibility of the WH domain is most likely a unique structural feature of DrRecQ. In contrast, the orientation of the WH domain in EcRecQ is very similar in both the free and nucleotide-binding structures. In addition, Swan et al. recently solved the crystal structure of BLM [20], which observes the mobility of its WH domain when comparing structures of apo- and DNA-bound forms. On the basis of the structural analysis, they also suggest that multiple open conformations exist in the apoform of BLM. The attribute is also consistent with our structural analysis of DrRecQ. Collectively, these findings provide the first evidence of interdomain flexibility in DrRecQ and the structural basis for a model of the conformational change in the WH domain that is likely to regulate DNA-binding activity. Further studies are necessary to elucidate the possible roles of the interdomain flexibility of the WH domain in DrRecQ function.

## 4. Conclusion

In this study, we described crystal structures of the DrRecQ helicase catalytic core and its ADP-bound form. Structural comparison of the two structures found that rigid-body movement occurs in the WH domain, indicating that the WH domain displays interdomain flexibility in DrRecQ. The motion may be important for the remarkable DNA repair mechanism performed by DrRecQ. Because DrRecQ is essential for maintaining genome stability, these structures provide important clues for understanding the molecular mechanisms of DNA replication, recombination, and repair.

## Figures and Tables

**Table 1:** Data collection statistics for the DrRecQ crystals. Values in parentheses are for the highest resolution shell.

	DrRecQ	DrRecQ-ADP
Data Collection		
Wavelength (Å)	0.9762	0.9762
Space group	P2_1_2_1_2_1_	P2_1_2_1_2_1_
Unit Cell (Å)	85.79, 98.52, 152.88	84.75, 95.61, 183.83
Resolution range (Å)	30–2.8 (2.9-2.8)	30–2.9 (3.0-2.9)
Total observations	86816 (9033)	345728 (34587)
Unique reflections	31511 (3115)	33811 (3294)
Completeness (%)	96.3 (96.6)	99.9 (100.0)
I/*σ*〈I〉	15.6 (2.9)	27.5 (3.8)
*R*_merge_ (%)	8.7 (54.3)	7.6 (67.8)
Refinement		
Resolution range (Å)	30–2.8 (2.9-2.8)	30-2.9 (3.0-2.9)
Reflections (F > 0 *σ*_F_)	31482 (3068)	33750 (3255)
*R*_cryst_ (%) for 95% data	21.3 (29.3)	22.2 (32.8)
*R*_free_ (%) for 5% data	28.5 (37.6)	29.5 (40.1)
RMS deviations		
Bond lengths (Å)	0.011	0.012
Bond angles (°)	1.58	1.78
Average *B*-factors (Å^2^)		
protein atoms	56.8	71.3
Zinc ion	36.1	66.4
ADP		72.8
Number of nonhydrogen atoms		
Protein	7839	7943
Zinc	2	2
ADP		54
